# Translation and cross‐cultural adaptation to Spanish of the Anterior Cruciate Ligament Return to Sport after Injury Scale (SP ACL‑RSI): Measurement properties and responsiveness in a multisport sample

**DOI:** 10.1002/jeo2.70046

**Published:** 2024-11-26

**Authors:** Rafel Donat‐Roca, Violeida Sánchez‐Socarrás, José M. Romero‐Sánchez, Salomé Tárrega, Tània Estapé‐Madinabeitia, Carles Escalona‐Marfil, Roberto Seijas, Georgia Romero‐Cullerés, Carolina Ochoa, Kate E. Webster

**Affiliations:** ^1^ Sport, Exercise and Human Movement (SEaHM) University of Vic–Central University of Catalonia Manresa Spain; ^2^ Faculty of Healthcare Sciences of Manresa University of Vic–Central University of Catalonia Manresa Spain; ^3^ Nursing and Physiotherapy Department, Faculty of Nursing and Physiotherapy University of Cádiz Cádiz Spain; ^4^ Research Group in Epidemiology and Public Health in the Digital Health Context (Epi4health) University of Vic–Central University of Catalonia Manresa Spain; ^5^ Instituto Cugat, Hospital Quirón Barcelona Spain; ^6^ Physical Medicine and Rehabilitation Department Althaia Xarxa Assistencial Universitària de Manresa Barcelona Spain; ^7^ Hospital Santa Caterina, Salt Girona Spain; ^8^ School of Allied Health, Human Services and Sport La Trobe University Melbourne Victoria Australia

**Keywords:** anterior cruciate ligament, rehabilitation, responsiveness, return to sport, Spanish ACL‐RSI, validation

## Abstract

**Purpose:**

The aim was to translate and adapt The Anterior Cruciate Ligament Return to Sport after Injury Scale (ACL‐RSI) to Spanish and provide evidence of its psychometric properties and responsiveness in a both sexes multisport sample.

**Methods:**

ACL‐RSI Spanish version (SP ACL‐RSI) was obtained by forward‐back‐translation method. Internal consistency, test‐retest reliability, construct validity and responsiveness were assessed. Standardized response mean (SRM), smallest detectable change (SDC) and minimally important change (MIC) were obtained by anchor‐based method. The sample consisted of *n* = 132 multisport patients who underwent ACL‐RSI. Sixty‐seven patients (Group A) completed test‐retest of the SP ACL‐RSI within 15 days and 65 patients (Group B) fulfilled SP ACL‐RSI, the Tampa Scale of Kinesiophobia (TSK‐11), the International Knee Documentation Committee‐Subjective Knee Form (IKDC‐SF 2000), the Knee Osteoarthritis Outcome Score (KOOS) preoperative, 6 and 12 months.

**Results:**

The SP ACL‐RSI shows satisfactory internal consistency (Cronbach's *α* = 0.95) and test–retest reliability (ICC = 0.92), with acceptable floor (9%) and ceiling (6%) effects. Convergent validity was supported with moderate positive correlations with KOOS and IKDC‐SF 2000 dimensions, and a negative correlation with the TSK (*p* < 0.001). For SDC responsiveness, a high effect was observed with SRM = 0.97 at 12 months, and the MIC for SP ACL‐RSI was 15.

**Conclusions:**

The SP ACL‐RSI is as valid and reliable as the original for measuring emotions, confidence in performance, and re‐injury risk on return to sport after ACL‐R in Spanish‐speaking multisport practitioners of both sexes. Moreover, it shows acceptable responsiveness, performing better at the group level than the individual level.

**Level of evidence:**

A cohort study (diagnosis); Level II of evidence.

**Abbreviations**
ACL: anterior cruciate ligament
ACL‐R: anterior cruciate ligament reconstruction
ACL‐RSI: Anterior Cruciate Ligament Return to Sport after Injury Scale
ACL‐RSI‐sp: Spanish version validated by Sala‐Barat et al in a soccer player sample
AUC: area under the receiver operating characteristic curve
BPTB: bone–patellar tendon–bone
ICC: Intraclass Correlation Coefficient
IKDC‐SF 2000: The International Knee Documentation Committee‐Subjective Knee Form
KOOS: The Knee Osteoarthritis Outcome Score
MIC: minimally important change
n: number of participants
PROMs: patient‐reported outcome measures
ROC: receiver operating characteristic
RTS: return to sport
SD: standard deviation
SDC: smallest detectable change
SEM: standard error of measurement
SP ACL‐RSI: Spanish version translated and validated in this current work.
SRM: standardized response mean
TSK‐11: Tampa Scale of Kinesiofobia 11‐item Short form

## INTRODUCTION

Easier access to sports has increased the number of people who practice sports, regardless of their physical condition [6]. At the same time, anterior cruciate ligament (ACL) injuries occur in increasingly heterogeneous groups of people, with differences in sex, age, sport, or level of competition being identified in recent years [18, 19].

Psychological factors secondary to ACL injury and surgical repair appear to play an important role in return to sport (RTS) [9, 22]. These factors, including reduced confidence and fear of re‐injury, can negatively impact recovery and the ability to return to pre‐injury competitive levels and performance [20, 23].

Among knee‐specific Patient‐reported outcome measures (PROMs), regarding the availability of instruments specifically developed to assess the ability to return to previous competitive levels and performance, the Anterior Cruciate Ligament Return to Sport after Injury Scale (ACL‐RSI) was developed specifically for this purpose [43]. Originally designed with three dimensions: emotions, confidence, and risk appraisal; subsequent analyses revealed that its 12 items should be treated as a uni‐dimensional scale [32]. The ACL‐RSI score serves as an objective measure of functional improvement following ACL reconstruction (ACL‐R) [3, 11]. Recognizing its clinical utility, the scale has been translated into 13 languages and validated accordingly [4, 5, 10, 13, 14, 15, 17, 28, 29, 30, 31], in some cases even, as in the case of Italian, resulting in two translations for the same language [36, 37].

In terms of its usefulness and applicability in both clinical and research settings, accurate information about the responsiveness of the ACL‐RSI could be crucial to assess its ability to detect significant change over time, both to guide clinical decisions and improve patient care, and to evaluate the impact of interventions and adapt rehabilitation strategies for better outcomes. Despite its importance, evidence on its responsiveness has been limited. Specifically, the non‐significant change offered by the Smallest Detectable Change (SDC), essential to distinguish true changes from measurement errors have been reported for the English, Swedish, Brazilian and Dutch versions; [17, 30, 31, 42] and the instrument's ability to explore, individually and in groups, the Minimally Important Change (MIC) perceived by the patient and reports are only available for the English and the Dutch version [32, 42].

Although a Spanish version has been previously published by Sala‐Barat et al (ACL‐RSI‐sp) [28], no evidence has yet been published on its responsiveness. In addition, despite the clinical value of the ACL‐RSI‐sp, its validation was performed in amateur and semi‐professional soccer players sample and objections to its translation and validation methodology suggest the need for a new Spanish version [21, 28]. This version should be applicable to a more diverse sporting population, including adequate representation of female athletes, ensuring cultural adaptation providing evidence on psychometric properties and clinical usefulness in RTS assessment [7, 21].

This study aimed to translate and adapt the ACL‐RSI to Spanish, and to provide evidence of its psychometric properties and its responsiveness in a both sexes multisport sample.

Our hypothesis is that the Spanish translation and adaptation will have similar psychometric properties to the original version.

## MATERIALS AND METHODS

A multicenter prospective cohort study (diagnostic) was performed.

### Translation and cross‐cultural adaptation

The internationally validated method of forward‐back translation was used to translate and adapt the ACL‐RSI to Spanish [39]. Two native bilingual linguists from the Language Department of our university independently translated the questionnaire from English to Spanish. The resulting versions were assessed by a bilingual team from the Faculty of Health Sciences, comprising an orthopedic surgeon specializing in ACL reconstruction, a biomechanics and sports science expert (RS), and a clinical psychologist specialized in chronic pain and cognitive‐behavioral therapy (TE‐M). After identifying and rectifying conceptual errors related to medical terminology, the initial draft of the Spanish version was generated. Later, two native English speakers, unaware of the original version, independently back‐translated from Spanish to English. Upon comparison with the original English ACL‐RSI version, a 90% similarity was achieved. To minimize semantic and conceptual differences between the Spanish and original versions, the final 10% underwent reformulation. This process involved consensus among the five bilingual translators and a physiotherapist expert in research methodology (CE‐M). Additionally, insights from 30 university students who had undergone ACL‐RSI in the past three years, as part of a pretest, were considered. The Spanish translation did not introduce any significant changes to the English version. As a minor adaptation, in questions 3, 7, and 9, the qualifier ‘mucho’ was changed to ‘muchísimo’ (very much) to facilitate respondents in selecting an appropriate score for these items. This modification stemmed from input gathered from both bilingual experts and participants during the pilot test of the initial translation. Furthermore, this adaptation was made while respecting cultural norms that could be generalized to other Spanish‐speaking population. Ultimately, a consensus version unanimously approved by experts was established as the final SP ACL‐RSI (Table 1).

**Table 1:** Spanish translation of the ACL RSI (SP ACL‐RSI).

Escala de Retorno a la Práctica del Deporte después de una Reconstrucción del LCA (SP ACL‐RSI)												
Instrucciones: Este cuestionario pretende conocer la sensación de riesgo a volverse a lesionar la rodilla. Marque de 0 a 10 aquella opción que se acerque más a su situación actual.												
1‐ ¿Está seguro de que su rendimiento deportivo estará al mismo nivel que antes?												
***Nada Seguro***	**0**	**1**	**2**	**3**	**4**	**5**	**6**	**7**	**8**	**9**	**10**	***Totalmente Seguro***
	□	□	□	□	□	□	□	□	□	□	□	
2‐ ¿Le parece probable volverse lesionar la rodilla, si retoma la práctica deportiva?												
***Totalmente Probable***	**0**	**1**	**2**	**3**	**4**	**5**	**6**	**7**	**8**	**9**	**10**	***Totalmente Improbable***
	□	□	□	□	□	□	□	□	□	□	□	
3‐ ¿Le inquieta volver a practicar su deporte?												
***Me inquieta muchísimo***	**0**	**1**	**2**	**3**	**4**	**5**	**6**	**7**	**8**	**9**	**10**	***No me inquieta en absoluto***
	□	□	□	□	□	□	□	□	□	□	□	
4‐ ¿Está seguro de que su rodilla no fallará durante la práctica deportiva?												
***Nada Seguro***	**0**	**1**	**2**	**3**	**4**	**5**	**6**	**7**	**8**	**9**	**10**	***Totalmente Seguro***
	□	□	□	□	□	□	□	□	□	□	□	
5‐ ¿Está seguro de que puede practicar deporte sin tener que preocuparse por la rodilla?												
***Nada Seguro***	**0**	**1**	**2**	**3**	**4**	**5**	**6**	**7**	**8**	**9**	**10**	***Totalmente Seguro***
	□	□	□	□	□	□	□	□	□	□	□	
6‐ ¿En relación con su actividad deportiva, es frustrante tener que pensar en su rodilla?												
***Muy Frustrante***	**0**	**1**	**2**	**3**	**4**	**5**	**6**	**7**	**8**	**9**	**10**	***Nada Frustrante***
	□	□	□	□	□	□	□	□	□	□	□	
7‐ ¿Le da miedo recaer de su lesión de rodilla a causa de la práctica deportiva?												
***Muchísimo Miedo***	**0**	**1**	**2**	**3**	**4**	**5**	**6**	**7**	**8**	**9**	**10**	***Nada de Miedo***
	□	□	□	□	□	□	□	□	□	□	□	
8‐ ¿Confía que la rodilla aguantará bien cuando la someta a presión?												
***No Confío En absoluto***	**0**	**1**	**2**	**3**	**4**	**5**	**6**	**7**	**8**	**9**	**10**	***Confío Totalmente***
	□	□	□	□	□	□	□	□	□	□	□	
9‐ ¿Le da miedo lesionarse accidentalmente la rodilla durante la práctica deportiva?												
***Muchísimo Miedo***	**0**	**1**	**2**	**3**	**4**	**5**	**6**	**7**	**8**	**9**	**10**	***Nada de Miedo***
	□	□	□	□	□	□	□	□	□	□	□	
10‐ ¿La idea de una nueva intervención quirúrgica y la consiguiente rehabilitación le impide practicar su deporte?												
***Me la impide Siempre***	**0**	**1**	**2**	**3**	**4**	**5**	**6**	**7**	**8**	**9**	**10**	***No me lo impide Nunca***
	□	□	□	□	□	□	□	□	□	□	□	
11‐ ¿Se siente seguro respecto a su capacidad para rendir durante la práctica de su deporte?												
***Nada Seguro***	**0**	**1**	**2**	**3**	**4**	**5**	**6**	**7**	**8**	**9**	**10**	***Totalmente Seguro***
	□	□	□	□	□	□	□	□	□	□	□	
12‐ ¿Se siente tranquilo con relación a la práctica de su deporte?												
***Nada Tranquilo***	**0**	**1**	**2**	**3**	**4**	**5**	**6**	**7**	**8**	**9**	**10**	***Totalmente Tranquilo***
	□	□	□	□	□	□	□	□	□	□	□	

*Note*: Anterior Cruciate Ligament Return to Sport after Injury Scale (ACL‐RSI) translated into Spanish, validated and culturally adapted by Donat et al. (2024).

### Participants and procedures

**Figure 1:**
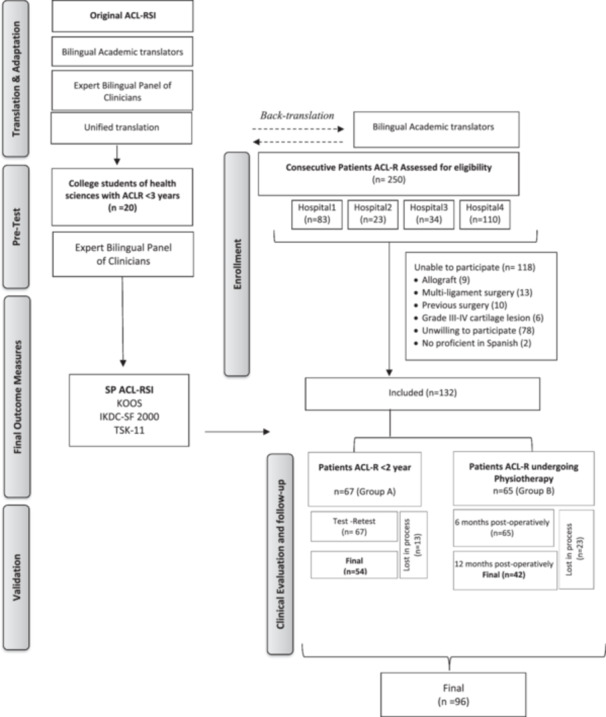
Study outline and participant flow diagram. ACL, anterior cruciate ligament; ACL‐R, anterior cruciate ligament reconstruction; SP ACL‐RSI, Spanish version of the Anterior cruciate ligament return to sport after injury; IKDC‐SF 2000, the International Knee Documentation Committee‐Subjective Knee Form; KOOS, Knee injury and Osteoarthritis Outcome Score; TSK‐11, Tampa Scale of Kinesiophobia.

Between 2015 and 2019, a total of 250 consecutive patients from four Spanish hospitals underwent an evaluation to determine their eligibility to participate in the study. Eligible candidates were those presenting with a unilateral ACL injury, with or without associated grade I or II meniscal and/or osteochondral lesions. Confirmation of ACL injury was established by two orthopedic surgeons by clinical examination. Patients with other knee injuries, previous knee surgeries, grade III or higher osteochondral lesions, arthritis, or those undergoing ACL allograft reconstruction were excluded. In addition, those without secondary education were excluded to ensure comprehension of the questionnaire. After applying the selection criteria, our initial sample comprised *n* = 132 participants who underwent ACL‐R, sample characteristics are shown in Table 2. These participants were divided into two groups: Group A consisted of individuals who had undergone ACL surgery within the past two years and completed their rehabilitation, while Group B comprised subjects with ACL rupture who had not yet undergone surgery. In Group A, the SP ACL‐RSI was administered twice within a 15‐day interval. Prospectively, Group B underwent preoperative and follow‐up assessments, during which participants completed the SP ACL‐RSI and other questionnaires. In the follow‐up of the participants, we scheduled the sending of reminders to answer the questionnaires every 5 days, considering no response within 15 days as a dropout from the study. The study outline and participants flow diagram are shown in Figure 1. At the end of the study, *n* = 96 participants remained.

**Table 2:** Demographic and clinical characteristics of the participants.

	Group A (*n* = 67)	Group B (*n* = 65)
Male sex, *n* (%)	43 (64.8)	48 (73.9)
Age (years), mean (SD)	26.5 (8.2)	27.7 (10.6)
Time from surgery (months), median (min, max)	15 (6, 23)	‐
Type of graft, *n* (%)		
BPTB	45 (67.2)	42 (64.6)
Hamstrings	22 (32.8)	23 (35.4)
Tegner Activity Score, median (min, max)	5 (1, 8)^a^	4 (2, 8)^b^
Hours of activity, median (min, max)	6 (0, 60)^a^	6 (1, 25)^c^

Abbreviations: BPTB, bone–patellar tendon–bone; *n* number of participants; SD standard deviation.

^a^ Assessed at baseline.

^b^ Assessed at 6 months.

^c^ Assessed at 12 months.

### Patient evaluation

To assess the validity of the SP ACL‐RSI, we administered Spanish‐validated versions of the Knee Osteoarthritis Outcome Score (KOOS) questionnaire and the International Knee Documentation Committee‐Subjective Knee Form (IKDC‐SF 2000), as a knee‐specific measures, along with the Tampa Scale of Kinesiophobia (TSK‐11) as a generic psychosocial measure [12, 16, 41].

The KOOS questionnaire was designed to identify the consequences of knee conditions in five dimensions: pain, symptoms, activities of daily living, sports and recreational activities, and knee‐related quality of life [27]. It consists of 42 items in a Likert format (0–5) and subscale scores are calculated as the sum of the items included. A higher total score (0–100) indicates greater functional independence.

The IKDC‐SF 2000 form was created to quantify patients' opinions affected by multiple knee injuries [16]. The instrument consists of 18 questions exploring three dimensions: symptoms, sports activity, and functionality. Since 2010, it has been used in different studies of Spanish‐speaking populations [1, 24, 25, 26].

The TSK‐11 [12] assesses activity avoidance and fear as well as fear of injury and pain. It consists of 11 questions scored on a Likert‐type scale (1–4). Higher scores indicate worse perceived outcomes [44].

The Tegner Activity Score assesses lifestyle and physical activity levels [33]. Scores (0–10) were considered to evaluate three levels: no activity, recreational activity, and amateur or professional activities, as well as the type of sport concerning the potential stress it could exert on the knee [2].

All questionnaires were administered using Limesurvey® software, version 2.00+ Build 131206.

### Statistical analysis

For descriptive analysis, categorical variables were summarized using absolute and relative frequencies. Quantitative variables were presented as means with standard deviations when the normal distribution assumption was met. When normality assumptions were not fulfilled, the median and range (minimum and maximum values) were reported. Normality was assessed using the Kolmogorov–Smirnov test.

In the assessment of measurement properties, each group had a distinct purpose. Specifically, Group A was examined for reliability: internal consistency, test‐retest, agreement whereas Group B underwent evaluation for construct validity, floor and ceiling effects, and responsiveness. A 95% confidence level was established for interval estimations. All analyses were conducted using STATA IC 17.

#### Reliability

Internal consistency for the SP ACL‐RSI total score was assessed using Cronbach's alpha. Test‐retest reliability (interrater reliability) was evaluated using the absolute‐agreement intraclass correlation coefficient (ICC). Agreement was assessed by calculating the standard error of measurement (SEM) computed as $\mathrm{SEM}=\mathrm{SD}\times \sqrt{1-\mathrm{ICC}}$, where SD is the standard deviation of the total score. The smallest detectable change (SDC) was defined as SDC$\;=1.96\times \sqrt{2}\;\times \;\mathrm{SEM}$. The percentage of individuals reporting an absolute difference between test and retest values exceeding the SDC was calculated.

#### Construct validity

Regarding discriminant validity, an independent samples *t*‐test was conducted to compare means and analyze the relationship between SP ACL‐RSI scores and both sex and graft type. Spearman's correlation coefficient was used to analyze convergent validity, as associations between SP ACL‐RSI scores and age, as well as with KOOS, IKDC‐SF 2000, TSK‐11, and Tegner Activity Score. The presence of floor and ceiling effects was assessed for each dimension of the SP ACL‐RSI questionnaire, including the total score, by examining the percentage of individuals reporting the lowest or highest possible scores (10th and 90th percentile, respectively). A predetermined threshold of 15% was established for these effects [35].

#### Responsiveness

Responsiveness was assessed by computing the standardized response mean (SRM), calculated as the mean difference between baseline and follow‐up divided by the standard deviation of the difference. Median values and corresponding confidence intervals, based on a free distribution when the dimension or total score was not normally distributed, were utilized. The interpretation of SRM is as follows: an SRM of <0.5 indicates a low effect, 0.5–0.8 reflects a moderate effect, and >0.8 signifies a large responsiveness [8]. Following the methodology proposed by Webster et al. in 2021, the anchor‐based method was employed to distinguish between patients showing improvement or no change during the two assessments (at 6 and 12 months) [42]. Patients with a worsened outcome at the second assessment were excluded from the analysis of minimally important change (MIC). The dichotomized KOOS‐QOL knee confidence question was used as an anchor (external) criterion of confidence in sports resumption to assess whether the patient perceived the change to be important. Univariate logistic regression analysis was performed using the dichotomized KOOS‐QOL knee confidence question (“Improved confidence” and “No change”) and the change between ACL‐RSI assessments as predictor variables to determine the optimal MIC. SDC between 6‐month and 12‐month follow‐up measures were calculated at the individual (SDC _individual_) and group (SDC _group_) levels. SDC _group_ was calculated as ${\mathrm{SDC}}_{\text{group}}=\frac{{\mathrm{SDC}}_{\mathrm{individual}}}{\sqrt{n}}$. An SDC _group_ < MIC indicated good ability of the scale to detect clinically relevant changes at the group level, distinguishing them from measurement errors. SDC _individual_ > MIC suggests the limited ability of the scale to detect clinically relevant changes at the individual level. Using a Receiver Operating Characteristic (ROC) curve, the sensitivity, specificity, and misclassification were calculated for each potential MIC value, and the highest value of the Youden index (sensitivity + specificity − 1) was used determine the optimal MIC. The area under the ROC curve (AUC) represents the probability that the measure correctly discriminates between improved and non‐improved patients. AUC can be interpreted as the probability of correctly discriminating between improved and non‐improved patients. Possible values for the AUC were: 0.5 considered random, 0.7–0.8 as acceptable or moderate and 0.8–0.9 as excellent [34].

#### Sample size

It has been recommended a sample size of at least 50 patients as adequate to determine the MIC and a ratio of respondents per item of at least 5:1 [35, 39, 40]. Therefore, considering a 20% of losses in follow‐up, the sample size was set at *n* = 60.

## RESULTS

### Reliability

Internal consistency was adequate, with a Cronbach's *α* of 0.95 (lower limit 95% CI: 0.94). The ICC for test‐retest indicated excellent stability at 0.92 (ICC 95% CI: 0.86–0.95). Regarding agreement, the SEM was 5.53, small compared to total score SD (19.6), and the SDC was 15.3 and the proportion of patients with difference exceeding it was 7%.

### Construct validity

Regarding the analysis of the association between SP‐ACL‐RSI and other variables and measurements, no significant associations were found for sex (*d* = −1.9; *p* = 0.73), age (*r* = −0.23; *p* > 0.07) or graft type (*d* = 6.1; *p* = 0.23). The mean scores and standard deviations of KOOS, IKDC‐SF 2000, TSK‐11, and Tegner Activity Score, together with their correlation coefficients (*r*) with the SP ACL‐RSI score are shown in Table 3. Moderate positive correlations were found between ACL‐RSI and the KOOS dimensions ‐except for the KOOS Sport and Recreational dimension, where the correlation was low‐ and a moderate negative correlation with TSK. A non‐significant correlation was observed with the Tegner Activity Score.

**Table 3:** Spearman's correlations between SP ACL‐RSI scores and other measures.

	Mean (SD)	*r*	(95% CI)	*p*‐value
KOOS				
Symptoms	78.7 (13.9)	0.58	(0.39, 0.73)	<0.001
Pain	76.3 (18.0)	0.63	(0.45, 0.76)	<0.001
Activities of daily living	88.0 (12.0)	0.70	(0.54, 0.80)	<0.001
Sports and recreational	61.7 (15.7)	0.47	(0.26, 0.64)	<0.001
Quality of life	51.0 (17.7)	0.67	(0.51, 0.79)	<0.001
IKDC‐SF 2000	67.8 (13.1)	0.57	(0.37, 0.71)	<0.001
TSK‐11	27.0 (4.6)	−0.68	(−0.79, −0.53)	<0.001
Tegner Activity Score	4.8 (1.8)	0.12	(−0.12, 0.36)	0.325

Abbreviations: CI, confidence interval; IKDC‐SF 2000, International Knee Documentation Committee Subjective Form; KOOS, Knee Osteoarthritis Outcome Score; *n*, number of participants; *r*, Spearman's correlation coefficient; SD, standard deviation; TSK‐11, Tampa Scale Kinesiophobia.

### Floor and ceiling effect

The percentages obtained for floor and ceiling effects were less than 15%. A floor effect (score ≤ 38.33) was observed just in *n* = 6 participants (9.2%), while a ceiling effect (score ≥ 94.17) was observed in only *n* = 4 (6.1%). Hence, the proportion of patients below the 10th percentile or above the 90th percentile for all dimensions and the total score was considered acceptable.

### Responsiveness

The SP ACL‐RSI mean (SD) at six months and at 12 months, and results of responsiveness analysis using SRM are shown in Table 4, a large effect size at 12 months after surgery was found according to the SRM results.

**Table 4:** Responsiveness using SRM.

SRM			
6 months	12 months	Change	SRM
Mean (SD)	Mean (SD)	Mean (SD)	‐
52.0 (20.0)	70.9 (20.3)	17.8 (18.4)	0.97

Abbreviations: SD standard deviation; SRM, standardized response mean.

**Figure 2:**
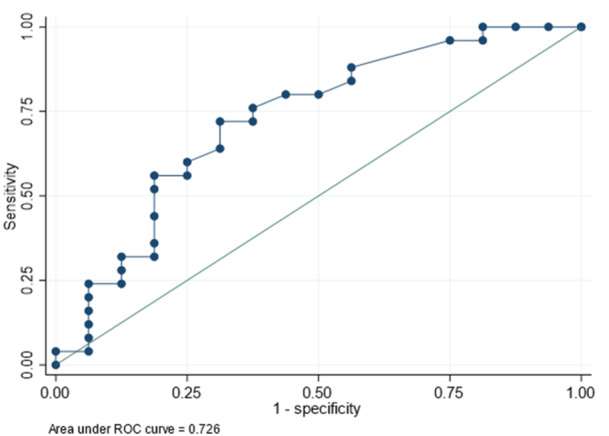
Receiver operating characteristic curve.

The maximum Youden value was 0.41 and MIC was stablished in 15. MIC was larger than SDC _group_ = 6.13 but lower than SDC _individual_ = 40.72. The 80% of individuals reporting a change greater than the MIC improved and 67% of individuals reporting a change smaller than MIC showed no change (Figure 2). According to the anchor‐based method, the area under the curve was moderate (AUC = 0.73) with a sensitivity of 72% and a specificity of 69%.

## DISCUSSION

This study successfully translated, cross‐culturally adapted and validated the ACL‐RSI into Spanish, obtaining an independent Spanish version of the one previously published by Sala‐Barat et al. [28], considering methodological objections raised [21], and employing the translation‐back‐translation method with a multisport sample comprising both sexes. Psychometric analyses support that the Spanish version of the ACL‐RSI has adequate reliability and validity. In addition, the responsiveness of the questionnaire was evaluated, demonstrating that it is a useful tool for measuring changes in rehabilitation in patients of various sports and of both sexes.

After the forward back‐translation process, the resulting SP ACL‐RSI version obtained was similar to Sala‐Barat et al.'s proposal [28]. However, notable differences emerged in writing style, as the obtained version employs a more formal language, addressing the reader as ‘usted’ rather than ‘tu’ (a style less used outside Spain). Moreover, SP ACL‐RSI refers to sports activity instead of soccer. Consequently, SP ACL‐RSI version, validated with diverse sports practitioners, holds the potential for application in a wider population compared to the original adaptation, which focused solely on federated soccer players.

Consistent with previous studies, the Cronbach's alpha of 0.95 demonstrated a high internal consistency and stands as the second‐highest among published versions, trailing only behind the French cross‐cultural validation [4]. However, it is noteworthy that a high Cronbach's alpha may imply redundancy in certain items of the instrument. This finding suggests the possibility of proposing a Spanish short version, based on a future factor analysis of the SP ACL‐RSI, akin to the approach adopted by Webster et al. for the original version [41]. Regarding test‐retest reliability, the ICC was adequate, akin to that observed in the Italian and Turkish versions [14, 37]. This outcome surpasses slightly those found in the Brazilian, Swedish, and original versions [17, 30, 43]. As for the test‐retest interval, in our study it was set at 15 days, in line with previous research. Although the timing of retesting was not specified in the original version of the ACL‐RSI [43], most studies performed it after a 2‐week interval [4, 5, 10, 13, 14, 15, 17, 28, 29, 31, 36, 37]. The Brazilian version was administered within five to eight days [30]. It has been argue that conducting a retest in less than eight days may enhance reliability; however, this effect could be attributed to recall [42].

As in previous studies, correlations between the ACL‐RSI and other questionnaires were evaluated to determine construct validity. Although there is no Spanish translation of the Tegner scale, it has been used in previous translations and cross‐cultural adaptations to Spanish [5, 13, 28, 29, 31, 36]. The results obtained in the SP ACL‐RSI (CI: ‐0.12, 0.36) were similar to those of the Dutch version [31]. Both studies presented the lowest values on this scale, which were slightly lower than those of other validations [5, 13, 29, 36]. The use of a heterogeneous population in terms of the type of sport practiced and competitive level of the sport would justify these results. Only the values of the ACL‐RSI‐sp version differed because the population used in its validation was strictly federated soccer players [28]. Concerning the questionnaires' used as outcome measures, the results of group B in the SP ACL‐RSI were associated to the IKDC 2000, since both questionnaires score lower when the handicap perceived by the respondent is higher. The correlation observed between the ACL‐RSI SP and the IKDC aligns with findings from other studies [10, 13, 30, 31]. Limited information is available on the Spanish version of the IKDC 2000 [25, 26]. While the IKDC 2000 is considered valid in patients with mixed knee pathologies and injuries, its evidence of validity in distinguishing clinically relevant changes in patients with ACL injuries is somewhat limited.

In contrast, the results observed for the SP ACL‐RSI and the Spanish version of the TSK in group B can be attributed to an elevated kinesiophobia score, potentially linked to a higher perception of impairment. The correlation results coincide with those reported in other cross‐cultural validations, especially in Chinese and Swedish populations [5, 17].

The floor and ceiling effects, below the widely accepted threshold of 15%, were minimal in line with the results of the Korean and Lithuanian versions [13, 29].

The Webster et al. revision of the original ACL‐RSI questionnaire reported a SEM of 9.6, with a recommended clinical SDC of 26.6 points [42]. While slightly higher than the SP ACL‐RSI and other versions, these variations may be attributed to study population heterogeneity, subtle translation differences, and cultural diversity [39]. Notably, SEM and SDC scores in our study align with those from the Dutch and Norwegian versions (5.5 and 15.3, respectively) [10, 31]. A test‐retest difference lower than the SDC value was reported in the present study.

The responsiveness was adequate and ranged from moderate to high using SRM. Webster et al. [42] found a moderately large ES (0.7), over the original version, by applying the questionnaires at the same temporality as in the present study. The moderate‐to‐large change in ES between 6 and 12 months may be attributed to an increased shift in the perception of injury risk during that period. Anticipating that patients were unlikely to return to sports six months post‐surgery, we expected psychological factors to normalize by 12 months [4]. Slagers et al. [32] administered the ACL‐RSI twice within a short two‐month span, reporting an ES of 0.3 in the English version. The brief interval between responses may influence values, as observed in the Dutch version [31]. Data suggest lower test sensitivity 6 months post‐intervention due to surgery's pronounced impact. After 12 months, patients' social determinants of health and self‐motivation became more influential, resulting in greater heterogeneity [38].

The anchored method allows the detection of clinically relevant changes that are important to patients [31, 42]. The overall MIC data for the study population conformed to values obtained by Webster et al. [42]. MIC results were superior to SDC values at the group level but not at the individual patient level.

### Limitations and strengths

The study is limited by a small sample for the assessment of responsiveness, even though a previous sample size calculation considered dropouts during follow‐up, which were higher than expected.

This study offers a versatile patient‐reported outcome measure (PROM) suitable for various sports. Notably, it is the second cross‐cultural validation, after the Dutch version, employing the anchor‐based method in the assessment of the ability of the ACL‐RSI to detect clinically relevant changes over time, including MIC.

## CONCLUSION

The SP ACL‐RSI is as valid and reliable as the original for measuring emotions, confidence in performance, and re‐injury risk on return to sport after ACL‐R in Spanish‐speaking multisport practitioners of both sexes. Moreover, it shows acceptable responsiveness, performing better at the group level than the individual level.

## AUTHOR CONTRIBUTIONS

Rafel Donat‐Roca was responsible for supervision of the study, the concept, design based on previous study and clinical experience of Kate E. Webster. Kate E. Webster is the lead author of the original version of the ACLRS. Violeida Sánchez‐Socarrás, and Kate E. Webster gave advice on the design of the study. Carles Escalona‐Marfil, Tània Estapé‐Madinabeitia were responsible of the forward back‐translation. Roberto Seijas, Georgia Romero‐Cullerés, and Carolina Ochoa collected the data. Salomé Tárrega and José Manuel Romero‐Sánche provided methodological support and analyzed the data. Rafel Donat‐Roca prepared the first draft of the manuscript, and all authors reviewed the first draft. Rafel Donat‐Roca and Salomé Tárrega wrote and modified the subsequent versions of the manuscript. All authors read and approved the final manuscript.

## CONFLICT OF INTEREST STATEMENT

The authors declare no conflict of interest.

## ETHICS STATEMENT

All participants provided written informed consent. The study protocol was approved by the Research Ethics Committee (CEIM) of the Fundació Unió Catalana d'Hospitals, CEIC 16/54.

## Data Availability

The data used in this study are available upon reasonable request to the corresponding author.
